# Evaluating the diagnostic accuracy of QIAreach QuantiFERON-TB compared to QuantiFERON-TB Gold Plus for tuberculosis: a systematic review and meta-analysis

**DOI:** 10.1038/s41598-024-65663-4

**Published:** 2024-06-24

**Authors:** Shima Mahmoudi, Sadra Nourazar

**Affiliations:** 1 Biotechnology Centre, Silesian University of Technology, Krzywoustego 8, 44-100 Gliwice, Poland; 2 Pediatric Infectious Disease Research Center, Tehran University of Medical Sciences, Tehran, Iran; 3 InPedia Association, Students’ Scientific Research Center, Tehran University of Medical Sciences, Tehran, Iran; 4 School of Medicine, Tehran University of Medical Sciences, Tehran, Iran

**Keywords:** QIAreach QuantiFERON-TB, QuantiFERON-TB gold plus, Diagnostic accuracy, Tuberculosis, Immunology, Microbiology

## Abstract

Accurate tuberculosis (TB) diagnosis remains challenging, especially in resource-limited settings. This study aims to assess the diagnostic performance of the QIAreach QuantiFERON-TB (QFT) assay, with a specific focus on comparing its diagnostic performance with the QuantiFERON-TB Gold Plus (QFT-Plus). We systematically reviewed relevant individual studies on PubMed, Scopus, and Web of Science up to January 20, 2024. The focus was on evaluating the diagnostic parameters of the QIAreach QFT assay for TB infection, which included sensitivity, specificity, positive predictive value (PPV), negative predictive value (NPV), positive likelihood ratio (PLR), negative likelihood ratio (NLR), and concordance with the QFT-Plus assay. QIAreach QFT demonstrated strong diagnostic performance with a pooled sensitivity of 99% (95% CI 95–100%) and specificity of 94% (95% CI 85–97%). Additionally, it showed a PLR of 15.6 (95% CI 6.5–37.5) and NLR of 0.01 (95% CI 0–0.03). The pooled PPV and NPV were 88% (95% CI 70–98%) and 100% (95% CI 99–100%), respectively. Concordance analysis with QFT-Plus revealed a pooled positive percent agreement of 98% (95% CI 88–100%) and pooled negative percent agreement of 91% (95% CI 81–97%), with a pooled overall percent agreement of 92% (95% CI 83–98). In conclusion, QIAreach QFT has shown promising diagnostic performance, with a strong concordance with QFT-Plus. However, further studies are needed to comprehensively evaluate its diagnostic performance in the context of TB infection.

## Introduction

Despite considerable advancements in preventing, diagnosing, and treating tuberculosis (TB), it persists as a significant global public health challenge. To effectively address this widespread problem, a comprehensive strategy integrating research, prevention, diagnosis, and treatment efforts is essential for a globally impactful approach to TB management^1^.

Over the past decade, the conventional understanding of TB has faced increasing challenges^2–4^. In recent years, there has been a growing demand for advanced point-of-care diagnostic assays driven by the need for rapid and simultaneous detection in diverse samples. These assays provide the unique advantage of delivering comprehensive and timely diagnostic information, making them invaluable tools across a spectrum of healthcare settings^5–7^.

Given the limitations of traditional Tuberculin Skin Tests (TSTs), an innovative diagnostic approach known as Interferon-Gamma (IFN-γ) Release Assay (IGRA) has been developed. The continuous evolution and enhancement of IGRA tests contribute significantly to global TB infection diagnostics, offering a more precise and targeted alternative to traditional TSTs^8,9^.

As of now, the World Health Organization (WHO) recommends three IGRA tests for detecting tuberculosis (TB). These include the T-SPOT.TB test by Oxford Immunotec, UK, QuantiFERON-TB Gold Plus (QFT-Plus) by Qiagen, USA, and Wantai TB-IGRA by Wantai, China. Moreover, the landscape of TB diagnostics is evolving with the introduction and development of several new assays. These include the QIAreach™ QuantiFERON-TB (QIAreach QFT) by Qiagen, USA, Standard E TB-Feron by SD Biosensor, Korea, LIOFeron TB/LTBI by LIONEX Diagnostics & Therapeutics GmbH, Germany, VIDAS™ TB-IGRA by bioMérieux, France, and AdvanSure™ TB-IGRA enzyme-linked immunosorbent assay (ELISA) by LG Life Sciences, Seoul, Korea. Additionally, three other IGRAs are currently in the development phase, namely ichroma™ IGRA-TB by Boditech Med Inc., Korea, T-Track® TB by Mikrogen GmbH, Neuried, Germany, and interferon protein-10 (IP-10) IGRA ELISA/lateral flow by rBioPharm, Germany^10,11^.

The lateral flow assay (LFA) is a versatile paper-based platform employed for the rapid detection and quantification of analytes within complex mixtures. This method involves placing the sample on a test device, with results typically visible within a short timeframe of 5–30 min. LFAs are characterized by their low development costs and ease of production, leading to widespread applications in various fields requiring rapid testing. LFAs accommodate a range of biological samples, including urine, saliva, sweat, serum, plasma, whole blood, and other fluids, making them adaptable to diverse diagnostic scenarios^5^.

The ichroma™ IGRA-TB assay^12^, and QIAreach QFT present promising prospects as point-of-care screening methods for the diagnosis of latent tuberculosis infection (LTBI). The QIAreach QFT test represents a noteworthy advancement in global TB infection diagnostics^13^. This innovative solution combines the advantages of IGRA technology with the convenience of a point-of-care test, making it suitable for use in non-traditional laboratory settings^14^.

The QIAreach QFT (QIAreach; QIAGEN GmbH, Hilden, Germany) is a semi-automated LFA that mimics the QFT-Plus mechanism. It measures IFN-γ levels in plasma released from CD4^+^ and CD8^+^ T-cells, utilizing a single blood collection tube (BCT) corresponding to the QTF-Plus TB2 tube^15^. Instead of employing the complex Enzyme-linked Immunosorbent Assay (ELISA) method for IFN-γ detection, QIAreach QFT utilizes digital fluorescence lateral flow nanoparticle technology. This technology condenses the IFN-γ detection process into a single cartridge (eStick) processed on a portable platform (eHub), providing a binary result (positive/negative) within 20 min^16^.

The emergence of new tests for TB infection underscores the need for a comprehensive review of the diagnostic landscape^11^. Identifying any gaps in this context is essential for facilitating the development and adoption of these assays. This review aimed to assess the diagnostic accuracy of QIAreach QFT for TB infection compared to the QFT-Plus.

## Methods

We adhered to the guidelines outlined in the Preferred Reporting Items for Systematic Reviews and Meta-Analyses (PRISMA) for conducting diagnostic tests systematic reviews and meta-analyses^17^. The protocol was published on PROSPERO (CRD42024508008).

### Search method

We systematically searched for relevant individual studies on PubMed, Scopus, and Web of Science until January 20, 2024, without restricting language or time. The following search terms were utilized:

- Tuberculosis” [Title/Abstract] OR “TB”[Title/Abstract] OR “mycobacterium tuberculosis infection” [Title/Abstract] OR “quantiferon tb gold plus” [Title/Abstract] OR “QFT-Plus” [Title/Abstract] OR “QFT”[Title/Abstract] AND “LFA” [Title/Abstract] OR “lateral flow assay” [Title/Abstract] OR “QIAreach” [Title/Abstract] OR “QIAreach QuantiFERON-TB assay” [Title/Abstract].

We explored the grey literature through Google and Google Scholar. Furthermore, we identified additional studies by examining the references cited in the selected papers. Relevant articles linked to the provided keywords were subsequently included in our analysis.

### Study selection

Initially, titles and abstracts were screened to identify papers suitable for further examination. Subsequently, two reviewers (SM and SN) independently conducted a thorough assessment of the full texts of the chosen publications to determine eligibility. Any discrepancies were resolved through consensus.

Inclusion criteria included studies meeting the following conditions: (1) use of QFT-Plus for detecting TB infection/disease; (2) availability of data on positive or negative results using both QFT-Plus and QIAreach QFT test.

SM and SN independently evaluated study eligibility, with disagreements resolved through consensus. Studies were eligible for inclusion in the analysis only if they provided comprehensive details in a binary classification format, as represented by a 2 × 2 table(s) showcasing true-positive, false-positive, true-negative, and false-negative results; and/or the availability of data on positive, negative, and overall agreement.

Exclusion criteria were applied based on: (1) inclusion of only positive QFT-Plus individuals at baseline; (2) utilization of QIAreach QFT test compared Standard F TB-Feron FIA Assay.

### Data extraction

Two researchers (SM and SN) independently acquired the following information from each study: initial author, country, publication year, sample size, demographic details such as sex and age of participants, participant numbers, counts of positive and negative results for both tests, and values for true-positive, false-positive, true-negative, and false-negative. Furthermore, data on positive, negative, and overall agreement were collected.

### Quality assessment

To assess the methodological quality of eligible studies, we employed the Assessment of Diagnostic Accuracy Studies (QUADAS-2) tool^18^. Specific yes/no signaling questions were generated for each QUADAS-2 domain.

### Statistical analysis

The analysis was conducted using Stata (version 14; Stata Corporation, TX, USA). Data from individual studies were pooled using a random-effect model to derive values for various measures of test accuracy, including sensitivity, specificity, positive likelihood ratio (PLR), negative likelihood ratio (NLR), positive, negative, and overall agreement, diagnostic odds ratio (DOR) with corresponding 95% CI. A hierarchical summary receiver operating characteristic (HSROC) model was constructed to summarize the overall diagnostic accuracy. Additionally, the calculation of Positive Predictive Value (PPV) and Negative Predictive Value (NPV) provided further insights into the diagnostic accuracy of the test across the included studies. Concordance analysis between the QIAreach QFT and QFT-Plus diagnostic assays was performed, evaluating Positive Percent Agreement (PPA), Negative Percent Agreement (NPA), and Overall Percent Agreement (OPA).

Heterogeneity was assessed using the *I*^*2*^ statistic, with *I*^2^ > 0.75 indicating high heterogeneity^19^. Deeks’ funnel plot was used to visually assess publication bias, with a *P* value ≤ 0.10 indicating significant asymmetry, thereby suggesting the presence of bias.

## Results

**Figure 1:**
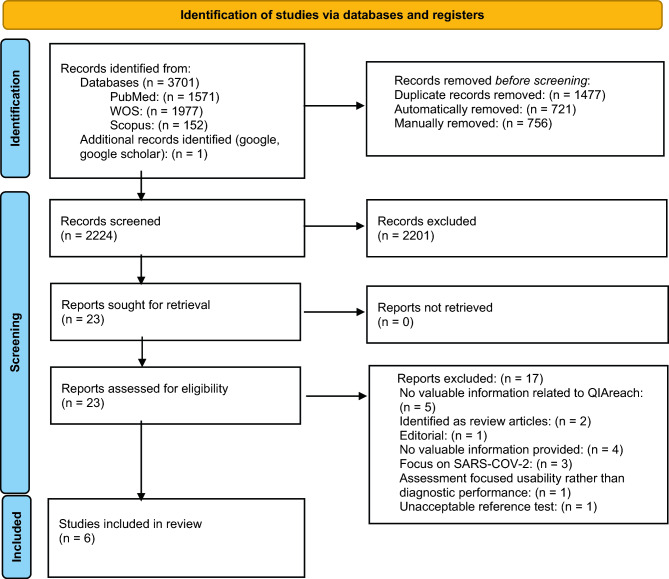
Study selection.

Figure 1 provides an overview of the selection process for included studies. Initial searches in databases yielded 3701 citations, and after removing duplicates, 2224 unique articles remained. Following the screening of titles and abstracts, 23 papers were selected for full-text reviews, and ultimately, six of those articles met the inclusion criteria for this study^15,20–24^. One study was excluded from the analysis due to its focus on the comparison of STANDARD F TB-Feron FIA vs. QIAreach QFT, which did not align with the criteria set for inclusion in the study^25^. In the Ntshiqa et al*.* study, our access was restricted to abstracts from a poster presentation. We had only results for true positive (*tp*) and true negative (*tn*) outcomes, along with information on the agreement of the test^24^.

### Characteristics of the included studies

Table 1 provides a summary of selected studies. These studies were conducted in different countries such as the USA^15^, Japan^21^, Italy^23^, Malaysia^22^, Vietnam^20^, Lesotho, South Africa, and Tanzania^24^. Saluzzo et al*.* evaluated the clinical performance of QIAreach QFT in detecting TB infection in the HIV-negative population with microbiologically confirmed pulmonary TB and healthy low-risk volunteers^23^. Fukushima et al*.* focused on adult patients with active pulmonary TB, alongside healthy low-TB-risk participants^21^. Vo et al. included individuals aged 18 or older meeting specific inclusion criteria related to TB exposure, comorbidities, and socioeconomic circumstances in the high TB burden country^20^. Ntshiqa et al*.* targeted household contacts of TB patients in three high-burden countries^24^, while Stieber’s research encompassed individuals aged 18 or older with and without varying risk factors for TB infection^15^.

**Table 1:** Characteristics of the included studies.

Author	Year	Study design	Country	Age (median [IQR])	Sex (male)	
					n	%
Fukushima et al*.*^21^	2019–2020	Cross-sectional	Japan	Active TB: 82 [76, 89], Healthy/low-TB-risk: 39.5 [30.75, 47.25]	37	45
Stieber et al*.*^15^	2021	Cross-sectional	USA	50 [25–71]	61	60
Saluzzo et al*.*^23^	2021	Cohort	Italy	–	–	–
Aziz et al*.*^22^	2021	Prospective trial	Malaysia	31 [25.75–41.25]	73	37
Ntshiqa et al.^24^	2021–2022	Cross-sectional	Lesotho, South Africa and Tanzania	27 (13–45)	163	35
Vo et al.^20^	2023	Cross-sectional	Viet Nam	61 [54–67]	97	37

### The diagnostic performance of QIAreach QFT

Table 2 presents an overview of the diagnostic performance of QIAreach QFT across diverse studies. In Ntshiqa’s study, a moderate concordance of QIAreach QFT against QFT-Plus was reported^24^. In this study, among household contacts with paired results, 42% (197 out of 465) tested positive, and 34% (156 out of 465) tested negative on both assays. Additionally, 24% (112 out of 465) had discordant results between the two assays.

**Table 2:** Diagnostic performance of QIAreach QFT across different studies.

Author	N	tp	fp	fn	tn	PPV			NPV			PPA			NPA			OPA		
						n	N	%	n	N	%	n	N	%	n	N	%	n	N	%
Stieber et al.^15^	206	68	6	3**	129	68	74	91.9	129	129	100	68	68	100	129	135	95.6	197	206	95.6
Fukushima et al*.*^21^	83	41	1	0	41	41	42	97.6	41	41	100	41	41	100	41	42	97.6	82	83	98.8
Saluzzo et al*.*^23^	304	120	7	0	177	120	127	94.5	177	177	100	120	120	100	177	184	96.2	297	304	97.7
Aziz et al*.*^22^	178	55	7	2	114	55	62	88.7	114	116	98.3	55	57	96.5	114	121	94.2	169	178	94.9
Vo et al*.*^20^	261	65	54	1	141	65	119	54.6	141	142	99.3	65	66	98.5	141	195	72.3	206	261	78.9
Ntshiqa et al.^24^	465	197	–*	–*	156	–	–	–	–	–	–	197	255	77.3	157	195	80.5	353	451	78.3

*tp* true positives, *fp* false positives, *fn* false negatives, *tn* true negatives, *PPV* positive predictive value, *NPV* negative predictive value, *PPA* positive percent agreement, *NPA* negative percent agreement, *OPA* overall percent agreement.

*The sum of false positives (*fp*) and false negatives (*fn*) in the study amounts to 112.

**3 were QFT-Plus-indeterminate results with Nil tube values > 10 IU/mL.

The comparative evaluation of QIAreach QFT demonstrates varying specificity across studies, ranging from 72 to 98%. This indicates differences in the test’s ability to accurately exclude individuals without TB in diverse research settings. Regarding sensitivity, the range spans from 96 to 100%, demonstrating high performance in correctly identifying individuals with TB across the studies. The comparative evaluation of QIAreach QFT demonstrates varying specificity across studies, ranging from 72 to 98%. This indicates differences in the test’s ability to accurately exclude individuals without TB in diverse research settings.

**Figure 2:**
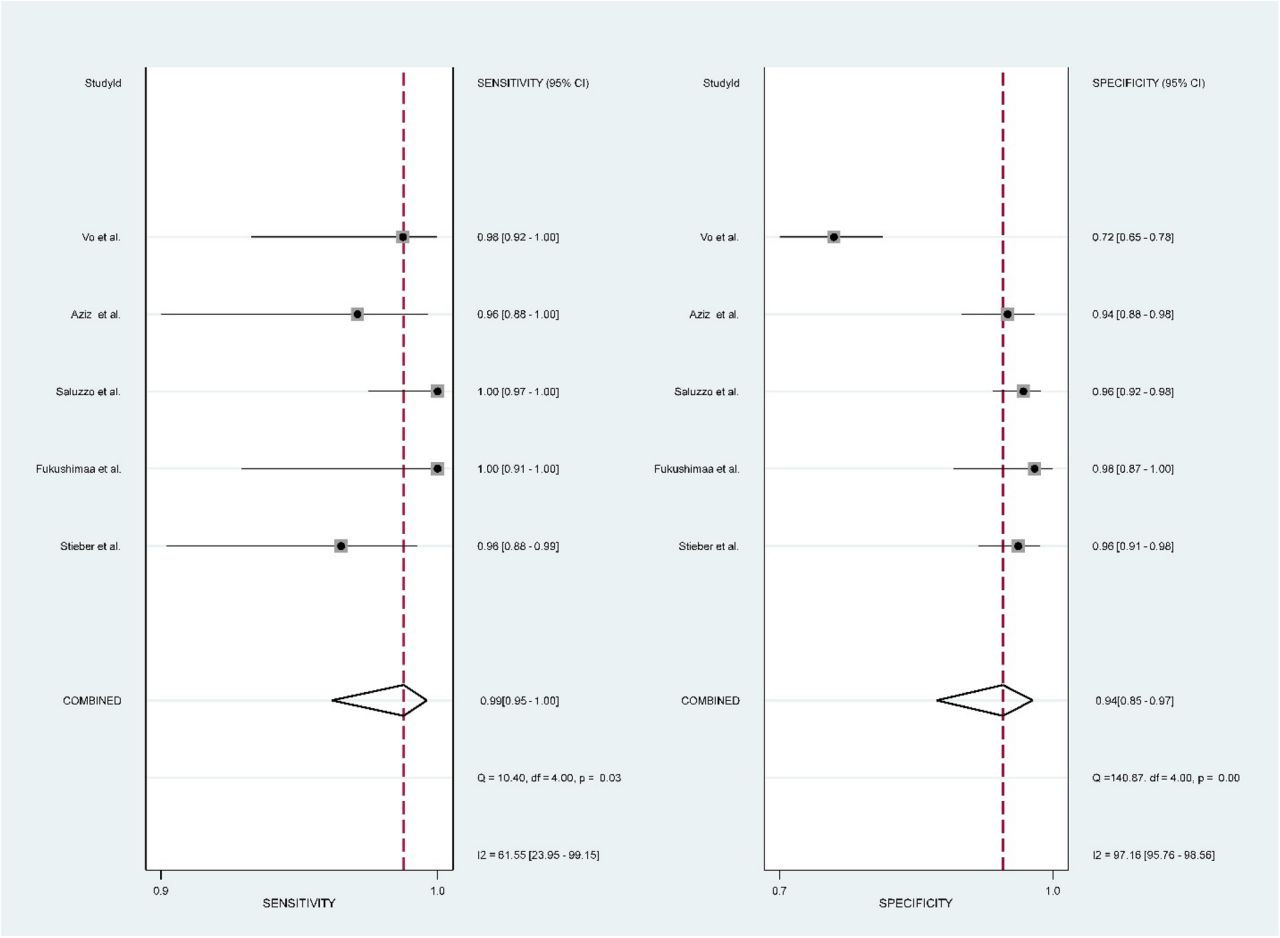
Pooled sensitivity and specificity along with 95% CIs for TB detection using QIAreach QFT.

**Figure 3:**
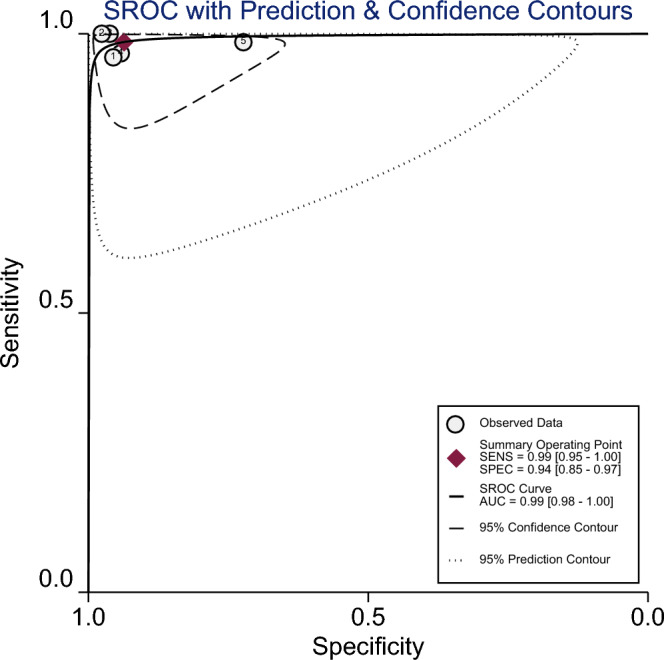
Summary receiver operating curve of the diagnostic performance of QIAreach QFT.

The analysis indicates that QIAreach QFT demonstrates a high pooled sensitivity of 99% (95% CI 97–100, *I*^2^: 61.55%), and a pooled specificity of 94% (95% CI 85–97, *I*^2^: 97.16%) (Fig. 2). Additionally, it demonstrated a PLR of 15.6 (95% CI 6.5–37.5) and a NLR of 0.01 (95% CI 0–0.03), suggesting its accuracy in confirming or excluding TB infections (Supplementary Fig. 1). The DOR was 1341 (95% CI 272–6602), reflecting the overall robustness of the test. The AUC of the SROC curve was 0.99 (95% CI 0.98–1.00) (Fig. 3).

**Figure 4:**
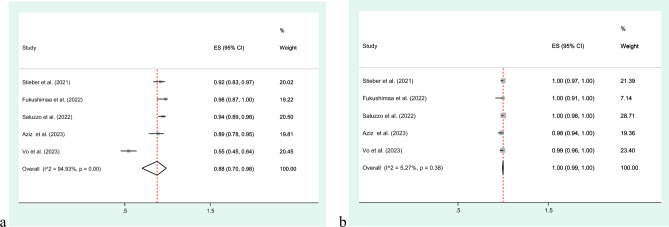
Pooled PPV and NPV along with 95% CIs for TB detection using QIAreach QFT; (**a**) PPV, (**b**) NPV.

The comparison of diagnostic performance among the studies reveals a range of values across key parameters. In terms of PPV, the studies exhibit a range from 54.6 to 97.6%, reflecting substantial variability in the ability of the tests to accurately identify true positive cases. NPV demonstrates a narrower range, from 98.3 to 100%, suggesting a high accuracy in ruling out true negative cases (Table 2). The pooled analysis showed a PPV of 88% (95% CI 70–98) and NPV of 100% (95% CI 99–100), respectively (Fig. 4).

**Figure 5:**
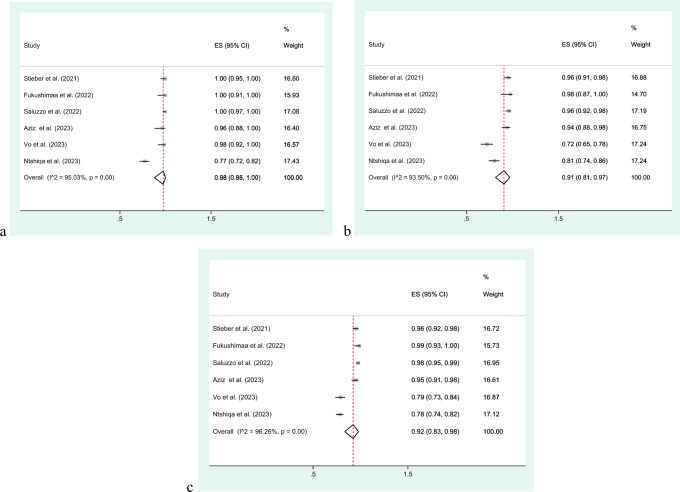
Concordance between the QIAreach QFT and QFT-Plus diagnostic assay: (**a**) Positive Percent Agreement (PPA); (**b**) Negative Percent Agreement (NPA); (**c**) Overall Percent Agreement (OPA).

In total, 6 studies, encompassing a participant pool of 1495 individuals, were incorporated to evaluate the agreement between the QIAreach QFT and the QFT-Plus diagnostic tests (Table 2). PPA varied from 77.3 to 100% across the studies. NPA across the studies demonstrated a range from 72.3 to 97.6%. The OPA values provide a comprehensive measure of the overall performance of the diagnostic tests across all participants in each study, considering both true positive and true negative results. OPA across the studies exhibited a range from 78.3 to 98.8%, emphasizing the diverse levels of agreement between the two tests. The meta-analysis demonstrated a pooled PPA of 98% (95% CI 88–100%) and pooled NPA of 91% (95% CI 81–97%), with a pooled OPA of 92% (95% CI 83–98) (Fig. 5).

The quality assessment of studies was conducted using QUADAS-2. According to the assessment, the risk of bias was considered unclear in four studies and high in three studies, primarily due to patient selection issues. Additionally, a high risk of bias was identified in four studies related to flow and timing issues. It’s noteworthy that applicability concerns were deemed low across all studies (Supplementary Fig. 2).

### Publication bias

**Figure 6:**
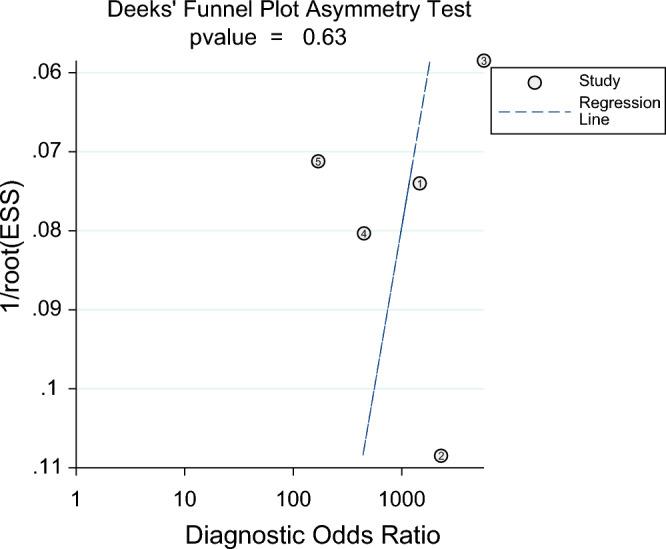
Deeks’ funnel plot asymmetry test for publication bias.

Publication bias was tested by linear regression, and Deeks funnel plot was drawn. The results showed no obvious publication bias (*P* = 0.63) (Fig. 6).

## Discussion

Our analysis revealed promising diagnostic characteristics through the comparative performance evaluation of QIAreach QFT and QFT-Plus. QIAreach QFT exhibits a high sensitivity of 99% (95% CI 97–100%), indicating its strong ability to correctly identify true positive cases. Additionally, its specificity of 94% (95% CI 85–97%) underscores the test’s capability to accurately recognize true negative cases. However, we observed relatively high heterogeneity (*I*^2^ = 97.16% for specificity) among the eligible studies. The PLR of 15.6 and NLR of 0.01 further emphasize the test’s effectiveness in confirming or ruling out TB infections. The DOR, calculated at 1341 (95% CI 272–6602), reinforces the overall robustness of the QIAreach QFT. In addition, the analysis revealed a pooled PPV of 88% (95% CI 70–98%), indicating the overall accuracy of the tests in correctly identifying individuals with true positive results across multiple studies. Conversely, the pooled NPV of 100% (95% CI 99–100%) emphasizes the high overall accuracy of the tests in excluding individuals correctly with true negative results.

The meta-analysis indicated strong concordance between the QIAreach QFT and QFT-Plus. It showed a PPA of 98% (95% CI 88–100%) for correctly identifying true positive cases and a pooled NPA of 91% (95% CI 81–97%) for accurately recognizing true negative cases. The Overall OPA was 92% (95% CI 83–98%). Discordant results in LTBI testing pose challenges in accurately categorizing individuals as either positive or negative, which can significantly impact subsequent clinical management decisions. Understanding the reasons behind discordant results is crucial for improving the reliability and accuracy of LTBI diagnostics. However, the absence of a definitive gold standard for LTBI complicates the interpretation of discordant results. To address this issue, further research and larger-scale studies are needed to establish a more standardized approach for interpreting LTBI test results.

The QIAreach QFT is an entirely new test, so independent evaluations of their sensitivity and specificity compared to other IGRAs are limited^26^. Most previous reports have compared QIAreach with QFT-Plus. Only one study has compared the performance of STANDARD F TB-Feron FIA with QIAreach QFT. While QIAreach QFT shows promising diagnostic performance, characterized by high sensitivity and specificity compared to QFT-Plus, its efficacy must be understood within the broader context of TB diagnostics and other IGRA tests.

A notable advantage of QIAreach QFT is its efficiency, offering rapid results within just 20 min. This expedites the testing process, making it more convenient and applicable across diverse clinical settings^20^.

On the other hand, QIAreach QFT, despite its benefits of providing standardized and automated results, encounters challenges related to blood volume requirements and potential interference, particularly seen in a strong interferon response in negative control tubes^27^. QIAreach QFT utilizes only one blood collection tube, lacking the negative (Nil) control, whereas QFT-Plus uses four tubes (TB1, TB2, Nil, and mitogen) for a thorough analysis. This discrepancy is particularly significant if QIAreach QFT positivity was calibrated using QFT-Plus thresholds^20^. Another concern arises from uncertainties and biases in interpreting test results when using QIAreach QFT, particularly compared to QFT-Plus, especially in cases with indeterminate QFT-Plus results and high IFN-γ response in negative control tubes.

IGRAs have become valuable tools for detecting LTBI, presenting advantages over traditional methods like the TST. However, it is crucial to acknowledge and address certain limitations associated with IGRAs, particularly in the context of LTBI diagnosis. One significant limitation is the incapacity of IGRAs to differentiate between latent TB infection and active TB disease. Additionally, immunocompromised individuals, such as those with HIV or undergoing immunosuppressive therapy, may experience reduced sensitivity in IGRA results.

Recognizing the urgent need for diagnostic tests with improved performance and predictive value in identifying individuals at risk of progressing to active TB, it is essential to evaluate the performance of TB infection tests across diverse risk groups^27^. The spectrum of TB encompasses a range of disease states, including minimal (non-infectious), subclinical (asymptomatic but infectious), and clinical (symptomatic and infectious)^2^. Understanding this spectrum is vital for comprehensive disease management. Evaluating this spectrum in different demographic and risk groups is essential to tailor effective interventions and improve outcomes. Addressing the specific diagnostic needs at different disease stages is crucial for effective patient management and treatment strategies. Recognizing the current gaps in existing diagnostic approaches can pave the way for the development and implementation of stage-specific diagnostic tools. Continuous monitoring of disease progression is an area where current tools may have limitations, highlighting the ongoing need for innovation and improvement in TB diagnostics.

Despite the promising results, this research has limitations. The heterogeneity in study characteristics, such as sample populations and study designs, may influence the generalizability of the findings. Additionally, the limited number of studies evaluating the QIAreach QFT underscores the need for more extensive research to establish its diagnostic capabilities comprehensively. Another notable limitation is the absence of a gold standard for LTBI diagnosis, complicating the evaluation of IGRA performance. In our study, we lacked information regarding the performance of QIAreach QFT in cases with indeterminate QFT-Plus tests. Therefore, caution should proceed, and further studies are necessary to validate its performance across diverse epidemiological settings and different population groups, both in low and high-burden countries.

## Conclusion

In conclusion, the QIAreach QFT demonstrated favorable diagnostic performance, with high sensitivity and specificity and a high concordance against QFT-Plus. However, the limited number of studies evaluating the QIAreach QFT indicates the need for further research to comprehensively understand its diagnostic capabilities in the context of TB infection.

### Supplementary Information

Supplementary Figures.

## Data Availability

The datasets used and/or analyzed during the current study are available from the corresponding author upon reasonable request.
