# Supplementary material for: Evaluating the diagnostic accuracy of QIAreach QuantiFERON-TB compared to QuantiFERON-TB Gold Plus for tuberculosis: a systematic review and meta-analysis
Source: Sci Rep. 2024 Jun 24;14:14455. doi: 10.1038/s41598-024-65663-4 (PMC11196697; doi:10.1038/s41598-024-65663-4)
Supplement: Supplementary file 1 — Supplementary Figures. [file 41598_2024_65663_MOESM1_ESM.docx]

Supplementary figure 1. Positive Likelihood Ratio (PLR) and Negative Likelihood Ratio (NLR) for TB detection using QIAreach QFT

| Study  **+**  **+**  **+**  **+** | Risk of Bias | | | |  | Applicability Concerns | | |
| --- | --- | --- | --- | --- | --- | --- | --- | --- |
| Patient Selection | **?**  Reference Standard | **+**  Index Test  Flow and Timing | **+** | **_**  Patient Selection |  | **+**  Index Test  0  Reference Standard | **+** | **+** |
| Stieber *et al.* | **_** | **+** | **+** | **?** |  | **+** | **+** | **+** |
| Fukushima *et al.* | **_** |  |  | **?** |  |  | **+**  **+**  **+**  **+**  **+** |  |
| Saluzzo *et al.* | **_** |  | **+**  **+**  **+** | **_** |  |  | **+** | **+** |
| Aziz *et al.* | **?** |  |  | **_** |  | **+**  **+**  **+**  **+**  **+** |  |  |
| Ntshiqa *et al.* | **+**  **+**  **+**  **+**  **+**  **+** |  |  | **_** |  |  |  |  |
| Vo *et al.* |  |  | **+** | **_** |  |  |  |  |

High Risk of Bias

**?**

**+**

Low Risk of Bias

Unclear Risk of Bias

**+**

**+**

**+**

Supplementary figure 2. Study quality assessment using QUADAS-2.
